# A Novel Method for Space Circular Target Detection in Machine Vision

**DOI:** 10.3390/s22030769

**Published:** 2022-01-20

**Authors:** Wenxue Hu, Jiannan Chi, Jiahui Liu, Zuoyun Yang

**Affiliations:** 1 School of Automation and Electrical Engineering, University of Science and Technology Beijing, Beijing 100083, China; liter_ustb@xs.ustb.edu.cn (W.H.); ustbljh@ustb.edu.cn (J.L.); b20160287@xs.ustb.edu.cn (Z.Y.); 2 Department of State Key Laboratory of Digital Manufacturing Equipment and Technology, Hua Zhong University of Science and Technology, Wuhan 430074, China

**Keywords:** computer vision, monocular vision, space circular target detection, noise resistance performance

## Abstract

Computer-vision-based space circular target detection has a wide range of applications in visual measurement, object detection, and other fields. The space circular target is projected into an ellipse in the camera for localization. Traditional methods based on monocular vision use a precise calculation model to calculate the center coordinate and normal vector of the space circular target according to the image’s elliptic parameters. However, this accurate calculation method has the disadvantage of poor anti-interference ability in practical application. Aiming at the shortcomings of the above traditional calculation method, this paper proposes an optimization method for fitting the circular target in 3D space, where the image ellipse is projected back into 3D space and then detects the center coordinate and normal vector of the space circular target. Unlike the traditional method, this approach is not sensitive to the image’s elliptic parameters; it has stronger noise resistance performance and notable application value. The feasibility and effectiveness of the proposed method were verified by both simulation and practical experimental results.

## 1. Introduction

Three-dimensional (3D) measurement and 3D reconstruction [1,2] are important research topics in the field of computer vision. According to the basic principle of computer vision, it is impossible to measure 3D space via monocular vision [3]. The measurement of 3D space points usually requires two or more cameras to form a binocular or multi-vision system [4]. For the purposes of certain applications, however, 3D measurement based on monocular vision is still a research hotspot [5]. A noise adaptive filtering structure was first explored in [6], where a new monocular vision feedback control strategy based on the adaptive filtering structure was developed for robotic positioning without noise parameters. Wang et al. [7] proposed a technique to measure the absolute depth information of the object in the image using monocular vision based on the Harris-SIFT corner detection algorithm. Researchers have established a 3D object detection and pose estimation based on monocular images [8], for example, where 3D object properties are first regressed using a deep convolutional neural network and then combined with geometric constraints provided by a 2D object bounding box to produce a complete 3D bounding box.

There have been many other valuable contributions to the literature. Xiang et al. [9] captured the 3D total motion of a target person from a monocular view input. Given an image or a monocular video, body, face, and finger motions could be reconstructed by a 3D deformable mesh model. This paper used 3D part orientation fields (POFs) to encode the 3D orientations of all body parts in a common 2D image space. The POFs were predicted by a fully convolutional network along with the joint confidence maps. An approach to monocular 3D human pose estimation and tracking was introduced in [10], which leveraged advances in reliable 2D pose estimation from monocular images, tracking-by-detection, and powerful modeling of 3D dynamics based on hierarchical Gaussian process latent variable models as a three-stage process to recover human poses in realistic street conditions.

Monocular vision depth estimation, which plays a crucial role in understanding 3D scene geometry, is an ill-posed problem. Recently, researchers have extensively investigated monocular depth estimation based on deep learning. Michels et al. [11], for example, applied supervised learning to learn monocular depth estimates in unstructured outdoor environments; the resulting depth estimates were sufficiently reliable to drive an RC car at high speeds through the environment. Monocular vision systems for 3D reconstruction and measurement are simple in structure, require relatively small quantities of data to operate, and have generally high processing speeds. However, their 3D measurement and reconstruction accuracy are inferior to multi-camera systems. It is still difficult to complete real high-precision 3D measurement tasks via monocular vision.

For certain rigid objects in space, monocular vision can indeed achieve accurate 3D measurement. For example, in calibrating camera parameters, the rigid transformation relationship between the camera frame and target frame can be obtained through the correspondence between spatial 3D primitives (e.g., points, lines) and graphic 2D primitives. In other words, monocular vision can be used to estimate the position and pose of rigid objects.

Monocular vision can also be used to accurately measure some specially shaped targets, such as rectangular target positions [12]. When the size of a space rectangular target is known, the center can be derived and accurately measured by monocular vision. Linnainmaa [13] and DeMenthon and Davis [14] also achieved the position estimation of space triangular targets with known sizes via monocular vision. The central position derivation and normal measurement of space circular targets are another common research topic. The projection of a circular target in space is generally an ellipse in the image. A location method of space conic targets was proposed in [15,16]; this method is not strictly limited to circular targets and does have a certain amount of versatility, but it is solved iteratively according to the initial value during computing, creating a multi-solution problem. D.H. Marimont et al. [17] proposed a space circular target measurement method accompanying a rigorous mathematical derivation. Eight groups of solutions corresponding to the center coordinate of the space circular target were gathered with a known radius, with four solutions in front of the camera and the others behind it. This method is also susceptible to redundant solutions. Kanatani and Liu [18] interpreted three 3D orthogonal lines by computing conics and then described an analytical procedure for computing the 3D geometry of a cone with a known shape from its projection. They also tested their method on real image examples. A space locating approach for space circular targets is presented in [19], where the center coordinate and normal vector of a space circle can be solved according to its image ellipse when its radius is known or unknown. Shiu and Ahmad [20] calculated the central position and normal direction of a space circular target and its image in the camera based on ellipse image features (e.g., major/minor axis of the image ellipse, ellipse center, angle between the long axis of the ellipse and the vertical direction). Generally, there are only two solutions for the calculation results. This method is well-suited to camera calibration and vision measurement. For example, a circular target with a known radius, when used as a calculation or measuring target, can realize the 3D position measurement of 3D space points via monocular vision.

Other researchers [21,22,23] have also analyzed computational errors in the process of space object reconstruction. Factors such as imaging distortion interference, camera inner parameter error, and feature target size [24,25] are the primary error sources in monocular-vision-based 3D target position and orientation measurement systems.

In summary, most existing space circular target location methods can accurately reveal the center and normal vector based on image characteristic parameters (which are related to the camera configuration) [19,20,26]. However, certain problems arise in the practical application of these methods. The corresponding algorithms depend on the space circular target and its elliptic projection in the image, so they are sensitive to image characteristic parameters. Subtle changes in image parameters (e.g., slight error in the long and short axes of the image ellipse, or in the angle between the long axis of the ellipse and the horizontal), for example, can cause a large deviation in the location of a space circular target [20]. The normal vector of a space circular target plane is only related to the posture of the image ellipse, but its center coordinate is relevant to the ellipse posture and circle radius. In practical applications, the radius of a space circular target is often already known, so the pose of the image ellipse is critical for accurate measurement of the space circular target.

It is necessary to detect the parameters of the imaging ellipse accurately during the image segmentation stage, but various noise factors alter the pose of the elliptic projection, thus creating measuring errors of the normal and center coordinates of the space circular target plane. When using this method for iris detection in an eye tracking system, it is dependent on (and sensitive to) the detection of image ellipse parameters. The anti-noise performance of the method proposed in [27] is relatively weak, so stringent requirements are necessary for any image segmentation application. This is highly challenging in most practical engineering scenarios, such as 3D gaze tracking, dynamic micro displacement measurement, and other fields.

This paper proposes a novel space circular target detection method based on monocular vision (also referred to here as “mono-vision”). The traditional calculation method is first used to perform segmentation and optimum fitting of the image elliptic projection to detect image ellipse parameters, then a 3D calculation model of the space circular target is built based on these parameters. Optimized parameters of the ellipse on the image are not extracted directly, but rather, edge points of the image ellipse are projected back into 3D space. The space circular target is fitted in this 3D space, then its center coordinate and normal vector are detected. Unlike the traditional method, this approach is not sensitive to image ellipse parameters; it has a strong anti-noise capacity and noteworthy potential application value. Simulation and experimental results verify the feasibility and effectiveness of the proposed method.

The rest of this paper is organized as follows. Section 2 provides a brief introduction to the traditional calculation method. The proposed method is described in Section 3. Section 4 presents simulation results, and Section 5 discusses the experiment conducted to further validate the proposed method. Section 6 contains a brief summary and concluding remarks.

## 2. Traditional Calculation Method

The perspective projection of a space circular target is an ellipse image in the camera. An accurate calculation method for the center coordinate and normal vector of a space circular target based on monocular vision is given in [19,20]. The method is implemented in the following steps.

### 2.1. Construction of the Cone Corresponding to Space Circular Target

**Figure 1:**
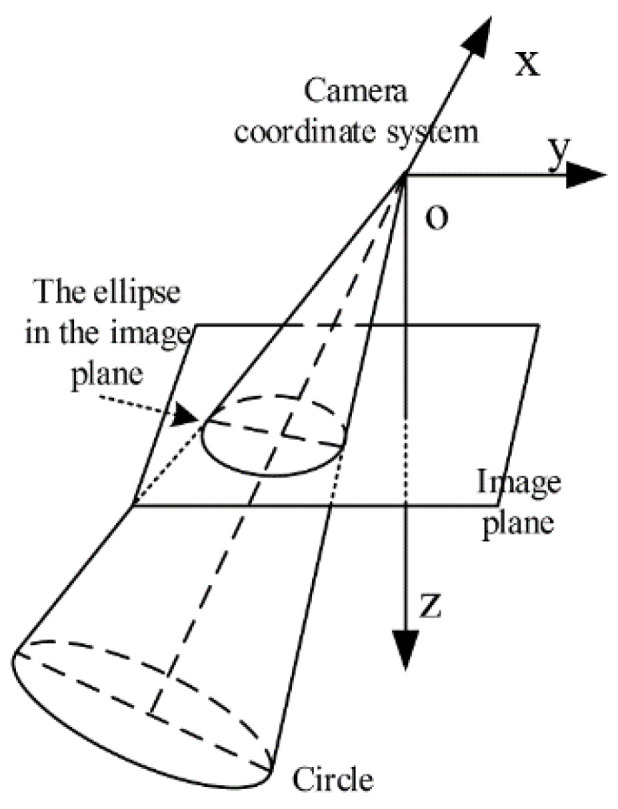
Perspective projection of a circular target.

As shown in Figure 1, according to the principle of pin-hole imaging, the edge of a space circular target, the center of a camera lens, and the edge of the ellipse image constitute a space elliptic cone.

#### 2.1.1. Space Vertebral Equation

According to graphics on the image interface, the elliptic equation fitted is $au^{2}+bv^{2}+cuv+du+ev+f=0$, and coefficients of the equation a-f are known. Each point (*u*, *v*) on the image ellipse becomes a line of the cone through (*u*, *v*, *f*_0_) and (0, 0, 0), where *f*_0_ is the focal length of the camera. Let the coordinate of the principal point be (0, 0), so the origin of the *u*-*v* frame is on the *z*-axis of the camera frame. The generatrix equation is $\frac{x}{u}=\frac{y}{v}=\frac{z}{f_{0}}$ and

(1) $$u=\frac{f_{0}\;x}{z},\;v=\frac{f_{0}\;y}{z}$$

By substituting Equation (1) into the fitted elliptic equation, the space vertebral equation is written as

(2) $$Ax^{2}+By^{2}+Cxy+Dxz+Eyz+Fz^{2}=0$$

where $A=af_{0}^{2}$, $B=bf_{0}^{2}$, $C=cf_{0}^{2}$, $D=df_{0}$, $E=ef_{0}$, F=f.

#### 2.1.2. Establishment of a New Frame

A new frame *ox*′*y*′*z*′ is created which has the same origin as the original frame; the vertebral equation is in a standard form in the new frame. Set a point (*x*, *y*, *z*) in the frame *oxyz*, the coordinate of which is (*x*′, *y*′, *z*′) in the frame *ox*′*y*′*z*′, so that (*x*, *y*, *z*)*^T^* = **P**(*x′*, *y′*, *z′*)*^T^*. The matrix **P** can be viewed as a rotation transformation matrix of the frame.

Standardize Equation (2) in terms of a quadratic form **Q**:

(3) $$[xyz]Q\left[\begin{array}{c} x\\ y\\ z \end{array}\right]=0,\mathrm{where}\;Q=\left[\begin{array}{ccc} A & \frac{C}{2} & \frac{D}{2}\\ \frac{C}{2} & B & \frac{E}{2}\\ \frac{D}{2} & \frac{E}{2} & F \end{array}\right]$$

In the new frame *ox*′*y*′*z*′, the *z*′-axis coincides with the axis of the space elliptic cone. The cone falls in the positive direction of the *z*′-axis. Assume $\left|\lambda_{1}\right|>\left|\lambda_{2}\right|$, then the major axis of the resulting ellipse is parallel to the *y*′-axis when using a plane parallel to the *x*′-*y*′ plane to intercept the cone.

After the quadratic form of the cone is standardized, the expression in the *ox*′*y*′*z*′ frame is

(4) $$\lambda_{1}{x^{\prime }}^{2}+\lambda_{2}{y^{\prime }}^{2}+\lambda_{3}{z^{\prime }}^{2}=0$$

Compare Equation (4) with the standard formula $\frac{{x^{\prime }}^{2}}{k_{x}^{2}}+\frac{{y^{\prime }}^{2}}{k_{y}^{2}}+\frac{{z^{\prime }}^{2}}{k_{z}^{2}}=0$ of a cone whose central axis is the *z*′-axis. In this case, $k_{x}=\rho \frac{1}{\sqrt{\lambda_{1}}}$, $k_{y}=\rho \frac{1}{\sqrt{\lambda_{2}}}$, $k_{z}=\rho \frac{1}{\sqrt{\lambda_{3}}}$, where ρ is any constant in $\lambda_{1}$, $\lambda_{2}$, $\lambda_{3}$, $\lambda_{3}$ and $\lambda_{1}$, $\lambda_{2}$ have opposite signs.

#### 2.1.3. Detection of Center Coordinate and Normal Vector of Space Circular Target

In the transmission projection cone of a circular target, two circles with radius *R* can be found by the intersection of a cross-section plane and the cone. When $\lambda_{1}>\lambda_{2}>0$, the space equation of the cross-section is ${x^{\prime }}^{2}\left(\frac{1}{k_{x}^{2}}-\frac{1}{k_{y}^{2}}\right)-{z^{\prime }}^{2}\left(\frac{1}{k_{y}^{2}}+\frac{1}{k_{z}^{2}}\right)=0$. Translating the section planes along the *z***′**-axis by 1 gives

(5) $$z^{\prime }=\pm \sqrt{\frac{\left|\lambda_{1}|-|\lambda_{2}\right|}{\left|\lambda_{2}|+|\lambda_{3}\right|}}x^{\prime }+1$$

**Figure 2:**
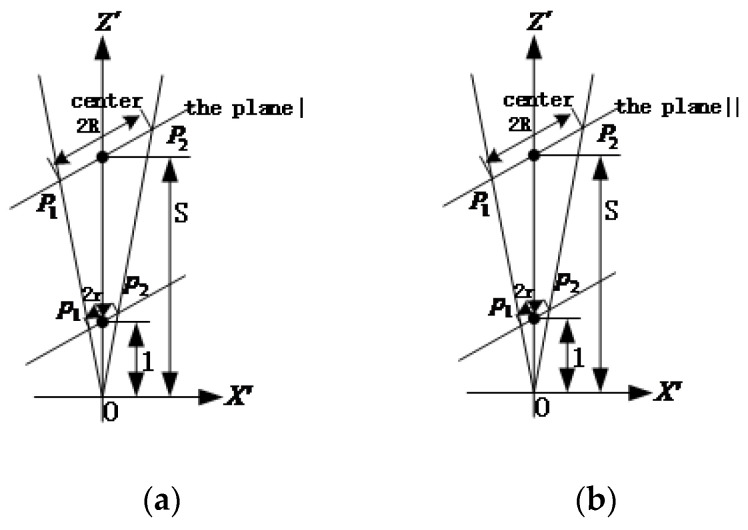
Cross-section plane and cone intersect in a circle with radius R. (**a**) Cross-section plane I; (**b**) Cross-section plane II.

The corresponding cross-section plane and the cone intersect in a circle with radius *r*. If the section plane is translated along positive direction of the $z^{\prime }$-axis by distance *S*, then *S* = *R/r*, as shown in Figure 2.

In the plane $x^{\prime }-z^{\prime }$, the cone is projected into two lines that are expressed as

(6) $$z^{\prime }=\pm \sqrt{\frac{\left|\lambda_{1}\right|}{\left|\lambda_{3}\right|}}x^{\prime }$$

According to Equations (5) and (6), ${x^{\prime }}_{p_{1}}=\frac{-1}{\sqrt{\frac{\lambda_{1}}{\lambda_{3}}}+\sqrt{\frac{\left|\lambda_{1}\right|-\left|\lambda_{2}\right|}{\left|\lambda_{2}\right|+\left|\lambda_{3}\right|}}}$,${z^{\prime }}_{p_{1}}=\frac{\sqrt{\frac{\lambda_{1}}{\lambda_{3}}}}{\sqrt{\frac{\lambda_{1}}{\lambda_{3}}}+\sqrt{\frac{\left|\lambda_{1}\right|-\left|\lambda_{2}\right|}{\left|\lambda_{2}\right|+\left|\lambda_{3}\right|}}}$; ${x^{\prime }}_{p_{2}}=\frac{1}{\sqrt{\frac{\lambda_{1}}{\lambda_{3}}}-\sqrt{\frac{\left|\lambda_{1}\right|-\left|\lambda_{2}\right|}{\left|\lambda_{2}\right|+\left|\lambda_{3}\right|}}}$, ${z^{\prime }}_{p_{2}}=\frac{\sqrt{\frac{\lambda_{1}}{\lambda_{3}}}}{\sqrt{\frac{\lambda_{1}}{\lambda_{3}}}-\sqrt{\frac{\left|\lambda_{1}\right|-\left|\lambda_{2}\right|}{\left|\lambda_{2}\right|+\left|\lambda_{3}\right|}}}$.

Radius *r* can then be calculated by

(7) $$r=\frac{\sqrt{{\left({x^{\prime }}_{p_{2}}-{x^{\prime }}_{p_{1}}\right)}^{2}+{\left({z^{\prime }}_{p_{2}}-{z^{\prime }}_{p_{1}}\right)}^{2}}}{2}=\frac{1}{\left|\lambda_{2}\right|}\sqrt{\frac{\left|\lambda_{1}\right|\left|\lambda_{3}\right|\left(\left|\lambda_{2}\right|+\left|\lambda_{3}\right|\right)}{\left|\lambda_{1}\right|+\left|\lambda_{3}\right|}}$$

The center coordinate of the circle with radius *r* in Figure 2a is ${x^{\prime }}_{o_{1}}=r\times \sqrt{\frac{\left|\lambda_{3}\right|\left(\left|\lambda_{1}\right|-\left|\lambda_{2}\right|\right)}{\left|\lambda_{1}\right|\left(\left|\lambda_{1}\right|+\left|\lambda_{3}\right|\right)}}$, ${y^{\prime }}_{o_{1}}=0$, ${z^{\prime }}_{o_{1}}=r\times \sqrt{\frac{\left|\lambda_{1}\right|\left(\left|\lambda_{2}\right|+\left|\lambda_{3}\right|\right)}{\left|\lambda_{3}\right|\left(\left|\lambda_{1}\right|+\left|\lambda_{3}\right|\right)}}$. If the inward unit normal vector of the circular target perpendicular to the circular plane is taken as negative, then ${v^{\prime }}_{1x}=\sqrt{\frac{\left|\lambda_{1}\right|-\left|\lambda_{2}\right|}{\left|\lambda_{1}\right|+\left|\lambda_{3}\right|}}$, ${v^{\prime }}_{1y}=0$, ${v^{\prime }}_{1z}=-\sqrt{\frac{\left|\lambda_{2}\right|+\left|\lambda_{3}\right|}{\left|\lambda_{1}\right|+\left|\lambda_{3}\right|}}$. Similarly, the center coordinate of the circle with radius R in Figure 2b is ${x^{\prime }}_{o_{2}}=-r\times \sqrt{\frac{\left|\lambda_{3}\right|\left(\left|\lambda_{1}\right|-\left|\lambda_{2}\right|\right)}{\left|\lambda_{1}\right|\left(\left|\lambda_{1}\right|+\left|\lambda_{3}\right|\right)}}$, ${y^{\prime }}_{o_{2}}=0$, ${z^{\prime }}_{o_{2}}=r\times \sqrt{\frac{\left|\lambda_{1}\right|\left(\left|\lambda_{2}\right|+\left|\lambda_{3}\right|\right)}{\left|\lambda_{3}\right|\left(\left|\lambda_{1}\right|+\left|\lambda_{3}\right|\right)}}$ and the normal vector coordinate of the circular target is ${v^{\prime }}_{2x}=-\sqrt{\frac{\left|\lambda_{1}\right|-\left|\lambda_{2}\right|}{\left|\lambda_{1}\right|+\left|\lambda_{3}\right|}}$, ${v^{\prime }}_{2y}=0$, ${v^{\prime }}_{2z}=-\sqrt{\frac{\left|\lambda_{2}\right|+\left|\lambda_{3}\right|}{\left|\lambda_{1}\right|+\left|\lambda_{3}\right|}}$. The results converted to the original camera frame oxyz are

(8) $$O_{1}^{T}=Po_{1}^{T},\;O_{2}^{T}=Po_{2}^{T}$$

where $P=\left[\begin{array}{cc} \begin{array}{cc} e_{1} & e_{2} \end{array} & e_{3} \end{array}\right]=\left[\begin{array}{ccc} e_{1x} & e_{2x} & e_{3x}\\ e_{1y} & e_{2y} & e_{3y}\\ e_{1z} & e_{2z} & e_{3z} \end{array}\right]$.

According to the above equations, the center coordinate *I* and unit normal vector **D** of the space circular target are

(9) $$I=r\left(\begin{array}{c} \pm e_{1x}\sqrt{\frac{\left|\lambda_{3}\right|\left(\left|\lambda_{1}\right|-\left|\lambda_{2}\right|\right)}{\left|\lambda_{1}\right|\left(\left|\lambda_{1}\right|+\left|\lambda_{2}\right|\right)}}+e_{3x}\sqrt{\frac{\left|\lambda_{1}\right|\left(\left|\lambda_{2}\right|+\left|\lambda_{3}\right|\right)}{\left|\lambda_{3}\right|\left(\left|\lambda_{1}\right|+\left|\lambda_{3}\right|\right)}}\\ \pm e_{1y}\sqrt{\frac{\left|\lambda_{3}\right|\left(\left|\lambda_{1}\right|-\left|\lambda_{2}\right|\right)}{\left|\lambda_{1}\right|\left(\left|\lambda_{1}\right|+\left|\lambda_{2}\right|\right)}}+e_{3y}\sqrt{\frac{\left|\lambda_{1}\right|\left(\left|\lambda_{2}\right|+\left|\lambda_{3}\right|\right)}{\left|\lambda_{3}\right|\left(\left|\lambda_{1}\right|+\left|\lambda_{3}\right|\right)}}\\ \pm e_{1z}\sqrt{\frac{\left|\lambda_{3}\right|\left(\left|\lambda_{1}\right|-\left|\lambda_{2}\right|\right)}{\left|\lambda_{1}\right|\left(\left|\lambda_{1}\right|+\left|\lambda_{2}\right|\right)}}+e_{3z}\sqrt{\frac{\left|\lambda_{1}\right|\left(\left|\lambda_{2}\right|+\left|\lambda_{3}\right|\right)}{\left|\lambda_{3}\right|\left(\left|\lambda_{1}\right|+\left|\lambda_{3}\right|\right)}} \end{array}\right)$$

(10) $$D=\left(\begin{array}{c} \pm e_{1x}\sqrt{\frac{\left|\lambda_{1}\right|-\left|\lambda_{2}\right|}{\left|\lambda_{1}\right|+\left|\lambda_{3}\right|}}-e_{3x}\sqrt{\frac{\left|\lambda_{2}\right|+\left|\lambda_{3}\right|}{\left|\lambda_{1}\right|+\left|\lambda_{3}\right|}}\\ \pm e_{1y}\sqrt{\frac{\left|\lambda_{1}\right|-\left|\lambda_{2}\right|}{\left|\lambda_{1}\right|+\left|\lambda_{3}\right|}}-e_{3y}\sqrt{\frac{\left|\lambda_{2}\right|+\left|\lambda_{3}\right|}{\left|\lambda_{1}\right|+\left|\lambda_{3}\right|}}\\ \pm e_{1z}\sqrt{\frac{\left|\lambda_{1}\right|-\left|\lambda_{2}\right|}{\left|\lambda_{1}\right|+\left|\lambda_{3}\right|}}-e_{3z}\sqrt{\frac{\left|\lambda_{2}\right|+\left|\lambda_{3}\right|}{\left|\lambda_{1}\right|+\left|\lambda_{3}\right|}} \end{array}\right)$$

## 3. Proposed Method

### 3.1. Mathematical Model of Space Circular Target Detection

A space circular target projects an ellipse in a camera [21]. In the proposed method, the edge of the elliptical target on the image plane is detected first. Based on edge points of the image ellipse, a space circular cone where the edge of the space circular target is located is created. A nonlinear equation system can be built to solve the center and normal vector of the space circular target by optimization according to the space geometry relation. The estimated solutions of the space circular target are then obtainable. Table 1 summarizes the notations used in this section.

**Figure 3:**
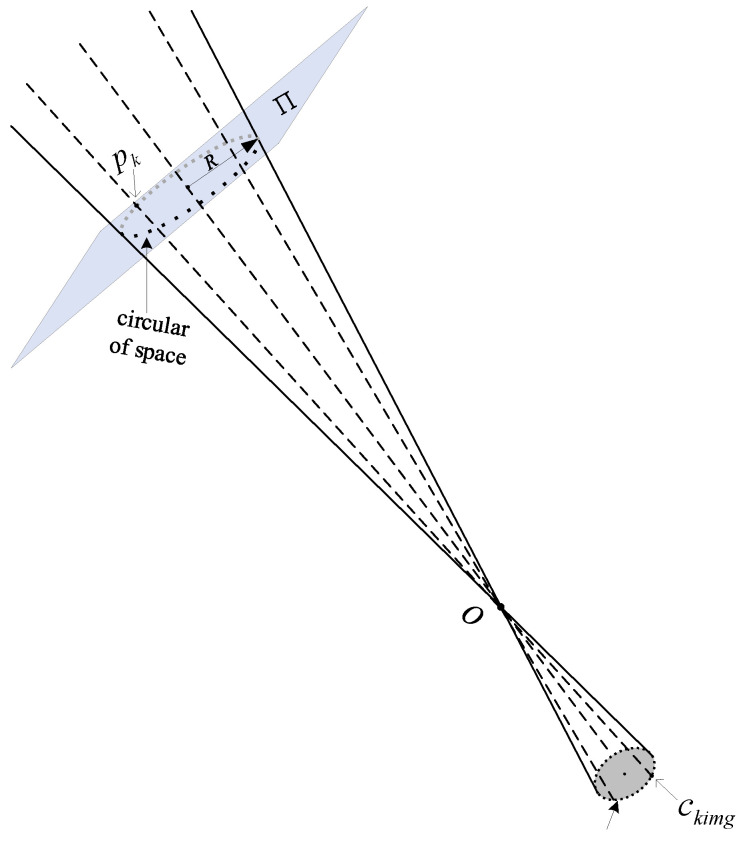
Back-projection of image elliptical contour points.

In Figure 3, **Π** is a space circular target plane and *O* is the optical center of the camera lens. Light emitted from edge points of the image ellipse is back-projected into 3D space through *O*. The mapped points together form several real space circular target planes. The radius *R* of the space circular target is known, so real space edge points can be fitted and the relation between the points can be expressed by a nonlinear equation system. The parameters of the space circular target can be roughly estimated after solving the equations.

There are numerous ellipse edge points on the image plane of the camera. In an actual image processing scenario, the elliptical contour on the camera imaging plane is sampled at an equal angle, then *N* image points of the elliptical edge are obtained. The image plane $C_{img}$ consisting of several sample points can be expressed as $C_{img}=(c_{1img},c_{2img},\cdots ,c_{kimg},\cdots ,c_{Nimg})$ accordingly, where *k* = 1, 2, …, *N*. The light from each sample point $c_{kimg}$ reaches a point $p_{k}$ on the space circular target plane Π through the optical center *O* of the camera lens, so the points $p_{k}$, *O,* $c_{kimg}$ are collinear. $p_{k}$ can be expressed by the following geometric relation:

(11) $$p_{k}=O+t_{k}(O-c_{k})$$

where $t_{k}$ is a scale factor that represents the length of vector $Op_{k}$.

Because $p_{k}$ is an edge point of the space circular target and the radius *R* of the space circular target is known, the relationship is expressed as follows:

(12) $$\left\|Pp_{1}\right\|=\left\|Pp_{2}\right\|=\cdots =\left\|Pp_{k}\right\|=\cdots =\left\|Pp_{N}\right\|=R$$

Additionally, *N* direction vectors $Pp_{k}$ composed of the edge point $p_{k}$ and center *P* of the space circular target are located on the space circular target plane **Π**. Thus,

(13) $$\left[Pp_{1}Pp_{2}Pp_{3}\right]=\left[Pp_{2}Pp_{3}Pp_{4}\right]=\cdots =\left[Pp_{k-1}Pp_{k}Pp_{k+1}\right]=\cdots =\left[Pp_{N-2}Pp_{N-1}Pp_{N}\right]=0$$

There are six unknown parameters in the nonlinear equation system formed from Equations (12) and (13), which may reveal the center *P* and normal vector n of the space circular target. An optimization algorithm can be applied to extract the space circular target.

### 3.2. Space Circular Target Parameter Detection Algorithm

Two parameters of the circular target including center and normal vector can be found according to the known radius *R* of the space circular target. First, from the elliptical edge coordinates on the image, a series of coordinate values are calculated in the system (camera) frame. The direction vectors $i_{k}$ of the line between them and the optical center of the camera are obtained through the mapping relationship.

**Figure 4:**
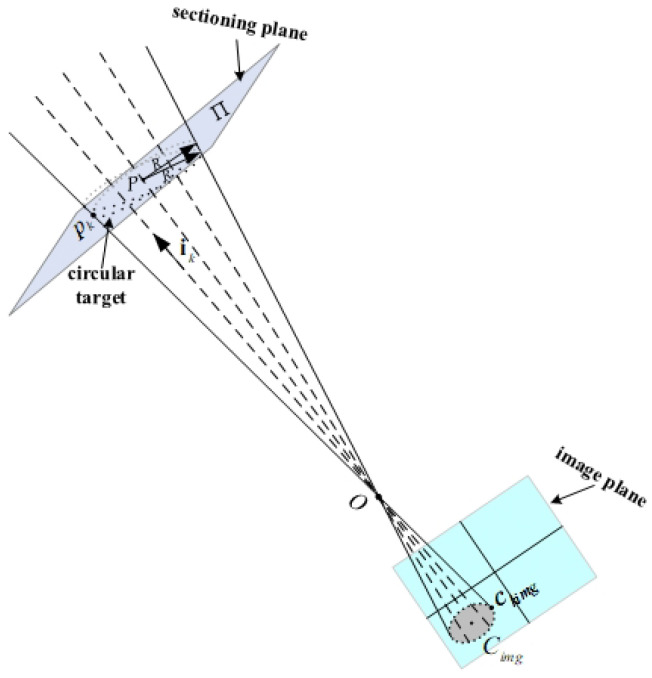
Parameter detection model for a space circular target.

As shown in Figure 4, *O* is the optical center of the camera, point $C_{img}$ is the center of the image ellipse, point *P* is the center of the space circular target whose normal vector is **n** and radius is *R*, and the direction vector of the line between an image elliptical edge point and *O* is expressed as $i_{k}$.

If any edge point of the image ellipse is assumed to be $c_{kimg}$, then for each $c_{kimg}$, the direction vector of the incident light $i_{k}$ is

(14) $$i_{k}=\frac{c_{kimg}O}{\left\|c_{kimg}O\right\|}$$

Each elliptical edge point $c_{kimg}$ on the image corresponds to the direction vector of an incident light $i_{k}$. The connection between any two points on the space circular target plane is perpendicular to the normal of the plane, so the satisfied equality is

(15) $$(p_{k}-P)\cdot n=0,$$

namely,

(16) $$n\cdot Pp_{k}=0$$

In Figure 4, the following relationship for three direction vectors **OP**, $Op_{k}$,$Pp_{k}$ composed of points *O*, *P*, $p_{k}$ is met:

(17) $$OP=Op_{k}+Pp_{k}$$

The following can be written after combining Equations (16) and (17):

(18) $$n\cdot OP=n\cdot (Op_{k}+Pp_{k})$$

and the simplified result is

(19) $$n\cdot OP=n\cdot Op_{k}$$

Figure 4 shows that $Op_{k}$ is $t_{k}i_{k}$, where $t_{k}$ is the length of two points *O*, $p_{k}$. Inputting $t_{k}i_{k}$ into Equation (19) provides the expression of the scale factor *t_k_*:

(20) $$t_{k}=\frac{n\cdot OP}{n\cdot i_{k}}$$

According to the above relation, for an edge point $p_{k}$ of any given space circular target, the optical center *O* of the camera and direction vector $i_{k}$ of the incident light satisfy the following expression:

(21) $$\begin{array}{l} p_{k}=O+t_{k}i_{k}\\ \begin{array}{cc} & =O+\frac{n\cdot OP}{n\cdot i_{k}}* \end{array}i_{k} \end{array}$$

while the space circular target plane consisting of *N* edge points satisfies the following relationship:

(22) $$\left\|p_{k}P\right\|=R$$

(23) $$\left[Pp_{k-1}Pp_{k}Pp_{k+1}\right]=0$$

In the linear equation system formed from 2*N* – 2 nonlinear elements of Equations (22) and (23), because the parameters to be solved are the center $P=[p_{x},p_{y},p_{z}]$ and normal vector $n=\left[n_{x},n_{y},n_{z}\right]$ of the space circular target including six unknown variables, when 2N−2≥6, so that N≥4, the calculation results are valid. If *N* = 4, there is a unique set of solutions. When *N* > 4, the nonlinear equation system is overdetermined. The data are mixed with model error or measurement noise, so it is generally impossible to determine a set of unknown parameter solutions uniquely to meet all 2*N* − 2 nonlinear elements of Equations (22) and (23). The least square method can be applied to remedy this.

Assume that the error vector is $E={\left[e_{1},e_{2},\cdots ,e_{k},\cdots ,e_{N},\cdots ,e_{2N-1}\right]}^{Τ}$, the first *N* items and items *N* + 1 to 2*N* − 1 of which can be expressed as

(24) $$E_{1\sim N}=\left\|p_{k}P\right\|-R$$

(25) $$E_{N+1\sim 2N-1}=[Pp_{k-1}Pp_{k}Pp_{k+1}]$$

The object function is:

(26) $$J=\sum_{i=1}^{2N-2}e_{k}^{2}=E^{Τ}E$$

Minimizing the square sum *J* of the error vector realizes the best estimation of the center *P* and normal vector **n** of the space circular target. The measured parameter values of the space circular target are ultimately obtained. The parameter calculation algorithm (Algorithm 1) is shown below.
**Algorithm 1:** Detection of the center *P* and normal vector **n** of the space circular target     **Input:** Radius of the space circular target *R*, optical center of the camera *O*, the number of edge points *C_kimg_* of the image ellipse m, error precision given eps, initial center *P_0_* of the space circular target, and random initial value **n**_0_ of the unit normal vector     **Output:** The optimal solution of center *P* and unit normal vector **n** of the space circular target      **Procedure:**
     1:         **for** k = 1 to m do     2: $i_{k}=O-c_{kimg}$$;\;p_{k}=O+\frac{n_{0}\cdot OP}{n_{0}\cdot i_{k}}*i_{k}$     3: $F_{1}(k)=\left\|p_{k}P_{0}\right\|-R;$     4: **end for**     5: **for** k = (*m* + 1) to (2 * *m* − 2) do     6: $F_{2}(k)=\left\|P_{0}p_{k-m}P_{0}p_{k-m+1}P_{0}p_{k-m+2}\right\|;$     7: **end for**     8: $J(P,\;n)=\sum_{k=1}^{m}F_{1}^{2}+\sum_{k=m+1}^{2m-2}F_{2}^{2};$     9: [*P*, **n**] = Index(Min(*J*));     10: **for** k = 1 to m do     11: $p_{k}=O+\frac{n\cdot OP}{n\cdot i_{k}}*i_{k};$     12: **if** k ≥ 2 then ≥     13: $V=\frac{Pp_{k}\times Pp_{k-1}}{\left\|Pp_{k}\times Pp_{k-1}\right\|};\;E=\mathrm{norm}(V-n);$     14: **end if**     15: **end for**     16: return *P*, **n**

## 4. Simulation Experiment

As discussed in this section, the proposed method was simulated to evaluate its feasibility and performance.

### 4.1. Simulation Environment

**Figure 5:**
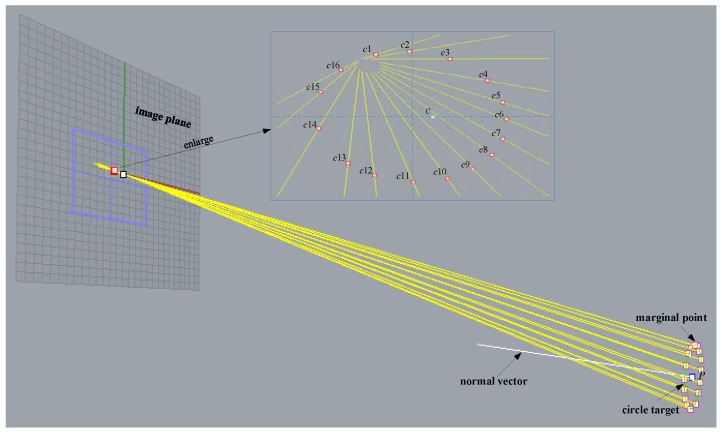
Simulation diagram of the space circular target mapping model.

The simulation experiment was conducted using 3D modeling software Rhinoceros 6.0 and Matlab 2016a. Rhinoceros is a powerful 3D modeling software, and Matlab is a mathematical software which is used in the fields of data processing and analysis. The simulation system was first built in Rhinoceros 6.0 according to the known model parameters. As shown in Figure 5, a frame was established with the camera imaging center as the origin whose coordinate is O=[0 0 0]. The *x*-axis and *y*-axis were created in the plane passing through the origin and parallel to the image plane, with the *z*-axis in the direction perpendicular to the image plane. This produced a space rectangular frame oxyz. The focal length of the camera was 16 mm and the radius of the space circular target was set to 6.5726701 mm. Uniformly spaced sampling was adopted to select 16 edge points of the space circular target for further simulation. All edge points were projected to the imaging plane through the optical center of the camera to obtain 16 elliptical edge points, then the image ellipse was obtained by fitting them.

### 4.2. Algorithm Feasibility

In general, a space circular target appears as an ellipse on the imaging plane of a camera; the connection between edge points of the image ellipse and optical center of the camera forms an elliptic cone. If a plane intersects the elliptic cone and the intercepted image is a circle with radius *R*, then the plane may fall into one of two positions. There are thus two solutions of the center and normal vector that may be obtained through imaging of the space circle.

The coordinates of the image elliptical edge points and radius *R* of the space circular target taken as input were substituted in this simulation into the detection procedure of the space circular objective parameters. For the given radius *R*, edge points of the image ellipse were mapped through the optical center of the camera *O*, and then two space circular planes were obtained with different directions and positions. A group of estimated values closest to the real values was selected artificially, and the other group was discarded automatically according to the known parameters in the simulation model. The resulting data are listed in Table 2.

The proposed algorithm was tested based on the input to obtain the estimated values of the center and normal vector of the space circular target (Table 2). The simulated and real values are in accordance, which indicates that the proposed method is feasible.

### 4.3. Algorithm Feasibility

The accuracy of the center and normal vector estimation of the space circular target is determined by a single factor: the image elliptical edge points (Section 3.2). In order to explore the influence of this factor on the parameters of the space circular target, Gaussian white noise whose signal-to-noise ratios (SNR) ranging from 100 to 40 were added to the edge points of the image ellipse at an interval of 10. Twenty measurement results were selected and averaged under each shifting condition. The resulting data are shown in Table 3.

For the same and certain elliptical edge pixels after adding Gaussian white noise, the space circular target parameters were calculated using the proposed method and the traditional method, respectively. The Euclidean distance and angle between vectors were used to represent the error between the real and estimated values of the target center and normal vector separately. The parameters were measured 20 times under each noise condition to obtain the measurement error values listed in Table 4 and Table 5. (Due to space reasons, only 10 sets of data are shown here).

**Figure 6:**
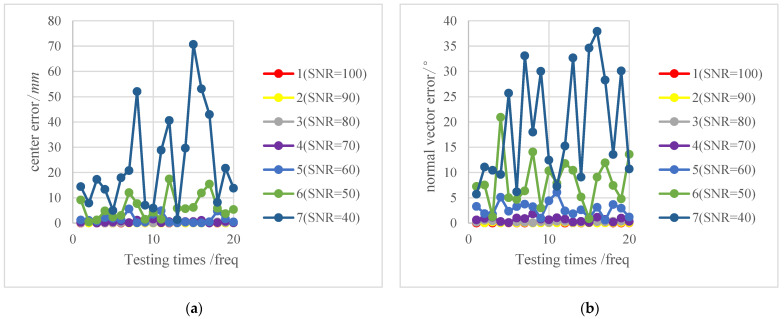
Error results of image elliptical edge points with noise. (**a**) Center error of the space circular target; (**b**) normal vector error of the space circular target.

Figure 6 shows the variation trends of the center and normal vector of the space circular target under different values of marginal noise. When the degree of noise is small (and the SNR is large), there was almost no interference to the target center and normal estimations; the fluctuation of the polyline tended to be stable. As the noise intensity increased, the influence of image elliptical edge points on the measured parameters increased gradually and the error value distribution grew irregularly.

The discreteness of measured parameters of the space circular target as well as the stability and concentrative level of data were assessed next. The average of 20 error values was calculated for each scenario to test the respective abilities of the two methods to solve the space circular target parameters.

The “solution effect” of the two methods is reflected in the parameter measurement errors under a consistent SNR distribution, as shown above. The estimation errors of the objective parameters are inversely proportional to the SNR. The robustness of the space circular objective parameter extraction results was evaluated next to further compare the proposed and traditional methods. As shown below, the error calculations of the two methods were placed on the same graph for an intuitive comparison.

**Figure 7:**
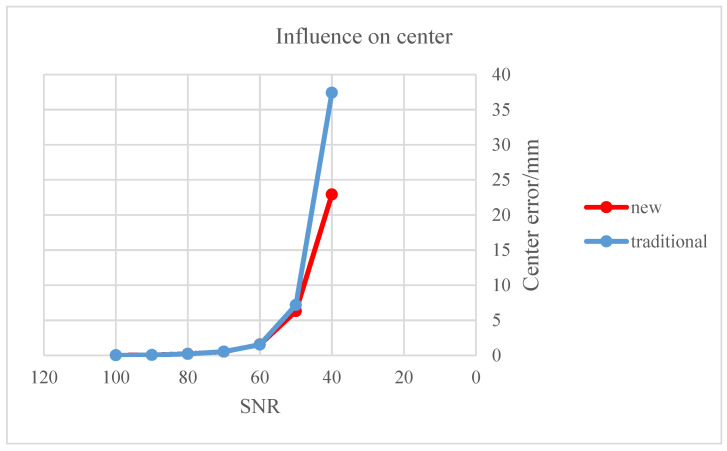
Influence of elliptical edge on center of space circular target.

**Figure 8:**
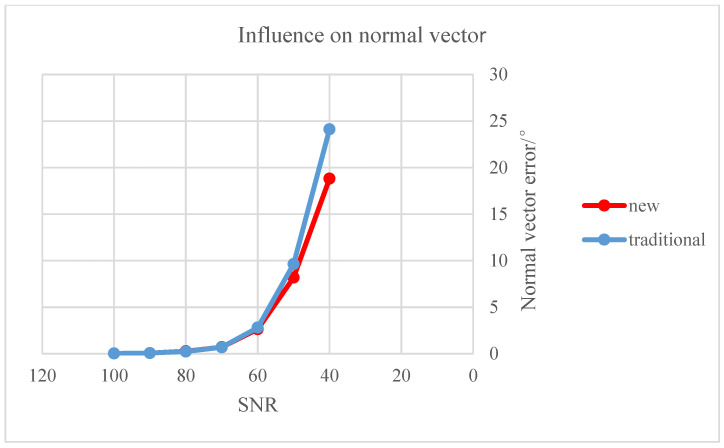
Influence of the elliptical edge on the normal vector of the space circular target.

Independent Gaussian white noise was added to the elliptical edge as position deviation of image elliptical edge points as the SNR was continually adjusted and the corresponding center and normal vector of the space circular target were calculated. The average error of the two methods for measuring these parameters is listed in Table 6; comparative graphs of their respective mean error are shown in Figure 7 and Figure 8. The red polyline marks the proposed algorithm and the blue polyline represents the traditional algorithm.

As shown in Figure 7 and Figure 8, the proposed algorithm has stronger anti-interference ability than the traditional algorithm. When the SNR is less than or equal to 60, the rising trend of the red polyline is gentler and the ascending height is significantly lower than the blue polyline. The traditional method produced maximum errors of the center and normal vector of the space circular target of about 37 mm and 24°, respectively; the maximum errors of the proposed algorithm were 23 mm and 19°, respectively.

## 5. Proposed Algorithm Application Example

The proposed method was used to reconstruct the optical axis of a human eyeball for 3D gaze estimation according to the center and normal vector of the iris. According to the human eye structure, the human iris can be regarded as a space circular target, and the optical axis of the eye can be represented by the normal vector of the iris. The better the optical axis is reconstructed, the more accurately will the final point-of-regard be estimated. The results were compared against those of a previously published optical axis and 3D gaze estimation method [28].

### 5.1. 3D Gaze Estimation Based on Center and Normal Vector of Iris

**Figure 9:**
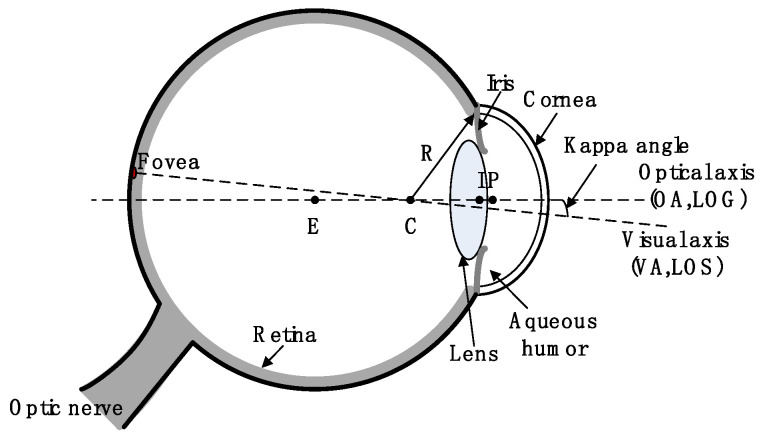
Human eyeball model.

3D gaze estimation methods work based on a geometric model and an imaging model of the eyeball [28]. Figure 9 shows a human eyeball model with a radius of about 12 mm. The sclera, iris, and pupil progress from the outer to the inner layer of the eyeball; the cornea covers the exterior of the iris. There is a ring in the center of the iris, the pupil, which adjusts the amount of light entering the eyes. The retina is located at the posterior wall of the eyeball. After entering the eyeball, light passes through a series of optical media, then reflects and refracts at various levels before reaching the retina.

A special area on the retina called the fovea contains a large amount of photoreceptor cells. The symmetry axis of the eyeball is the optical axis, where the fovea is not precisely located; it does form, however, a visual axis with the center of the cornea. There is a fixed angle between the optical axis and visual axis of the eyeball, which is called the kappa angle. 3D gaze estimation serves to estimate the direction of the visual axis. To align with the special structure of the eyeball, a 3D gaze estimation method usually involves first reconstructing the optical axis of the eyeball, and then converting it into the visual axis according to the specific kappa angles of the eye under analysis. Finally, points-of-view are calculated according to the position of the screen.

**Figure 10:**
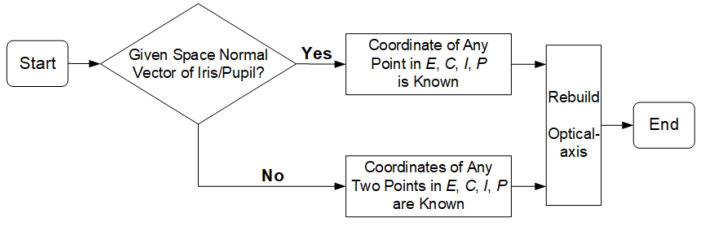
Human eyeball model.

The eyeball center *E*, cornea curvature center *C,* iris center *I*, and pupil center *P* are all located on the optical axis, which is always perpendicular to the plane where the margins of the pupil and iris are located. If the iris is regarded as a space circular target, the direction vector of the optical axis can be represented by the normal vector of the iris. Figure 10 shows various means of reconstructing the eyeball’s optical axis using visual features in a 3D gaze estimation system. In the case of the given space normal vector of the iris, if the coordinate of any point in *E*, *C*, *I*, and *P* is known, the optical axis can be reconstructed.

The center and normal vector of the iris were detected to reconstruct the optical axis of an eyeball in this experiment. This a two-step process: (1) user calibration and (2) 3D gaze estimation. User calibration serves to calibrate the iris radius and kappa angle [28], and then 3D gaze estimation estimates the optical axis and visual axis of the eyeball and points-of-regard on the screen.

In the user calibration stage, calibrations of iris radius and kappa angle were achieved using a previously published method [28]. The center and normal vector of the iris were then calculated by the proposed method to construct the optical axis of the eyeball. The visual axis of the eyeball was then constructed based on the optical axis and the calibrated kappa angle for visual direction detection. According to the position of the screen in the camera frame determined in the system calibration step [29], the intersection points between the visual axis and screen were calculated to determine the falling points of the user’s gaze.

**Figure 11:**
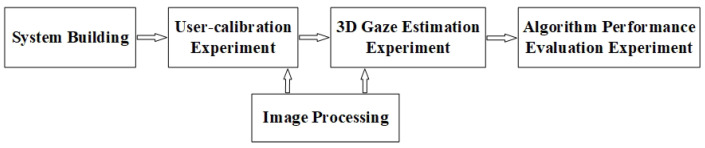
Experimental procedures on the actual system.

A flowchart of the experimental procedures run on the actual system, which mainly included user calibration and 3D gaze estimation, is shown in Figure 11. The 3D line-of-sight estimation results using the proposed method were compared against the results using the traditional calculation method.

### 5.2. System Estimation and Image Processing

#### 5.2.1. System Establishment

**Figure 12:**
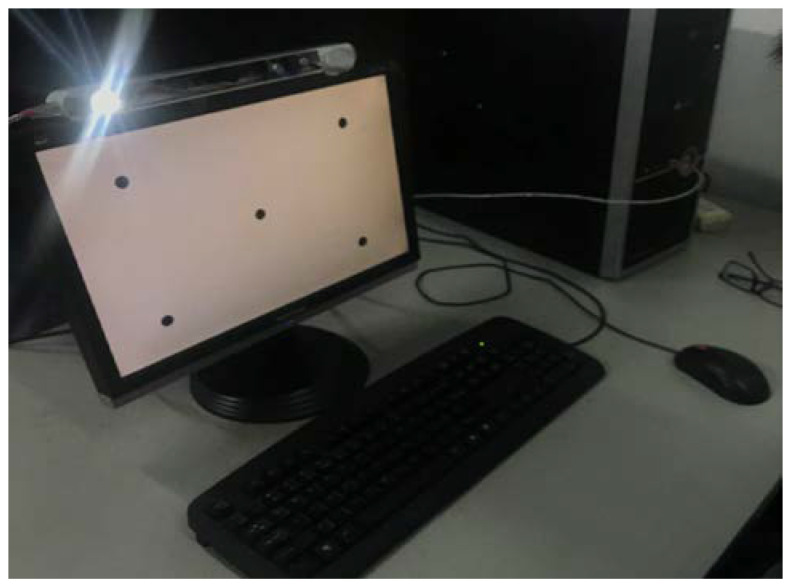
Construction of gaze tracking system.

As shown in Figure 12, a gaze-tracking system based on remote one-camera-one-single source is adopted to carry on the experiment. The gaze-tracking device is placed at the top of a computer screen. The camera is in the middle and the light source is on the left side of it. The focal length of the camera is 3.66 mm, the resolution is 640 × 480, and the pixel size is 2.2 μm. A source of visible light is chosen as the system illuminant in order to detect the iris margin more accurately. Before user calibration and 3D gaze estimation experiment, camera calibration and system calibration were carried out for the above system. Camera calibration is used to obtain internal parameters of the camera including lens focus, principal point coordinate, etc. System calibration is used to determine the position of the light source and the equation of the screen in the system camera frame.

#### 5.2.2. Eye Image Processing

It is necessary to detect eye images and extract visual features of the user’s eyeballs in the user calibration and gaze estimation processes for sound optical axis reconstruction. This includes detection of the iris edge, long and short axes of the iris ellipse, and other relevant elements. Eye image processing provided a workable basis in this study for validating the proposed method on an actual gaze-tracking system.

**Figure 13:**
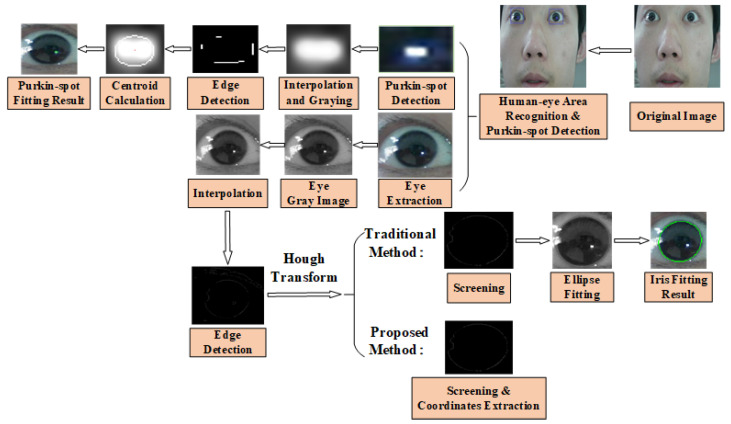
Iris detection and Purkin spot location.

As users gazed at the screen, the system camera captured images of their faces and eyes at a frequency of 30 Hz. Irises and bright spots were detected, respectively, in each frame of face images including eye localization, iris location, iris segmentation and edge detection, iris fitting, bright spot location, and sub-pixel extraction of the spot center. The iris and Purkin spot detection process is shown in Figure 13.

The iris edge was detected and subjected to ellipse fitting. The proposed algorithm uses edge points of the iris to detect its parameters; the traditional algorithm uses iris contours obtained by fitting.

### 5.3. User Calibration Experiment

As mentioned above, the general gaze estimation can be divided into two stages. The first step is user calibration. Structural parameters of the eyeball with individual differences are calibrated in this step. The user-calibrated ocular parameters in this experiment include the iris radius and kappa angle. As shown in Figure 12, five calibration points were set on the screen; then, each user sat 350–600 mm in the front of the computer screen and observed them sequentially. The system synchronously took images of the user’s face as they gazed at each point. The face images were processed through the method described in Section 5.2.2 to abstract the visual features of the eyeball including the iris edge points and iris center. Based on boundary points of the iris in the image, the method described in Section 3 was used to calculate the center and normal vector of the spatial iris to reconstruct the optical axis of the eyeball according to the calibrated iris radius.

The reconstructed optical axis of the eyeball was calibrated using two separate processes: (1) according to the iris radius calculated using the method of Ref. [29] and (2) according to the coordinates of the calibration points on the screen, with visual axes of the eye calculated as the user gazed at the calibration points. Then, the kappa angle between the optical axis and visual axis of the eyeball was calculated. User calibration was performed on each subject as recorded in Table 7.

The normal range of an iris radius is 5–6.8 mm and the kappa angle is about 5°. The calibrated iris radius and kappa angle identified in this experiment are within the normal limits.

### 5.4. 3D Gaze Estimation Experiment

An additional 3D gaze estimation experiment was performed after obtaining specific eye invariant parameters of the users through the calibration experiment. Nine test points were set on the screen and the system camera again took images of the user’s face as they gazed at each test point. The face images were processed by the method discussed in Section 5.2.2. The method presented in Section 3 was used to calculate the optical axis of the eyeball based on the boundary points of the iris. According to the fitting iris ellipse in the image, the traditional method of calculating the center and normal vector of the space circular target [28] was also used to reconstruct the optical axis of the eyeball. According to the optical axes reconstructed by the two methods and the kappa angles in the user calibration experiment, the visual axes and points-of-regard on the visual axes on the screen were calculated. Table 8 shows the RMSE estimation results of the two methods for different subjects’ points-of-regard in the *x* and *y* directions compared with the actual points on the screen.

Based on the calibrated parameters of the eyeball structure (iris radius), the proposed method and traditional method were separately used to calculate the center and normal vector of the space iris circle to construct the optical axis of the eyeball and calculate points-of-regard. As shown in Table 8, the proposed method calculated gaze points in the *x* direction more accurately than the proposed method; its accuracy in the *y* direction, however, was slightly lower. This demonstrates the effectiveness and feasibility of the proposed method in calculating the center and normal vector of the iris.

The main reason for the low accuracy of point-of-regard coordinates in the y direction was an iris edge point sampling problem. It was necessary in the experiment to extract several edge points from the detected boundary points of the iris to calculate the center and normal vector of the space iris circle. This extraction process generally depends on uniform sampling. Therefore, compared with the traditional method, edge information of the image iris was inevitably lost; in other words, using part of the edge points to calculate the center and normal vector of the space circular target is an incomplete process. Statistically speaking, the space circle detection process is more accurate when more uniform and abundant edge points are used, but this drives down the operation speed.

## 6. Conclusions

This work is oriented toward engineering applications. This paper proposed a new space circular target detection method which was designed to enhance the anti-interference ability of the traditional space circular target detection method. The image of a space circular target is an ellipse in the camera. Under the condition that radius R of the space circular target is known, a spatial cone is constructed via back-projection of the elliptical contour points. Based on the geometric relations satisfied by the target parameters, optimum fitting of the space circular target in 3D space can be realized. The center and normal vector of the space circular target can then be detected accordingly. This method is insensitive to image elliptic parameters, which gives it stronger noise resistance than the traditional method.

A simulation was conducted to evaluate the performance of the proposed algorithm. The proposed method was also tested in a 3D line-of-sight estimation experiment to determine its effectiveness and practical value. The proposed method was proved, in principle, to suppress noise interference in space circular target ellipse images more effectively than the traditional method. However, by comparing the running time of Matlab programs for the two methods, its calculation speed is relatively slow due to its space fitting technology, which is related to the number of image ellipse edge points. In the future, we plan to improve the speed of our algorithm while fully preserving its anti-interference performance.

## Figures and Tables

**Table 1:** Summary of notation.

Symbol	Meaning
**Π**	space circular target plane
*R*	radius of the space circular target
*P*	center of the space circular target
$p_{k}$	point on the plane **Π**
**n**	normal vector of the plane **Π**
*O*	optical center of the camera lens
**C** *_img_*	image plane
*c_kimg_*	image point of the elliptical edge
*C_img_*	center of the image ellipse
**i** *_k_*	direction vector of the incident light
**E**	error vector

**Table 2:** Feasibility simulation results of parameter extraction method.

Ordinal	Center/mm	Normal Vector (°)
Real-value	(−30.7587037, −99.5438179, 310.8944607)	(−0.1806, 0.1086, −0.9775)
Estimator	(−30.75871, −99.54382, 310.8944)	(−0.18064, 0.10861, −0.97753)

**Table 3:** Average space circular objective parameters after adding noise.

SNR	Average Target Center/mm	Average Target Normal (°)
100	(−30.75948, −99.54674, 310.90354)	(0.1805745, −0.1088265, 0.97522)
90	(−30.75983, −99.54666, 310.90386)	(0.180292, −0.108703, 0.9775875)
80	(−30.75167, −99.51862, 310.81855)	(0.1814295, −0.108838, 0.977349)
70	(−30.76763, −99.57437, 310.97225)	(0.17916, −0.10788, 0.97778)
60	(−30.66826, −99.22247, 309.85171)	(0.184879, −0.086483, 0.977705)
50	(−30.39077, −98.41919, 307.32605)	(0.1708489, −0.079914, 0.968743)
40	(−28.96868, −93.14112, 290.77975)	(0.025638, 0.0568438, 0.951424)

**Table 4:** Target center error results of image elliptical edge points with noise.

Center Error/mm		SNR						
		100	90	80	70	60	50	40
**Testing times/freq**	1	0.013478	0.1103	0.12109	0.37022	1.2358	9.1648	14.4498
	2	0.035129	0.027306	0.52507	0.5	1.0978	0.47822	7.9312
	3	0.018137	0.13744	0.12194	0.041915	0.77607	1.2489	17.3156
	4	0.011009	0.034212	0.029731	0.32756	2.1542	4.8409	13.3499
	5	0.018038	0.059334	0.10069	0.47824	1.8669	2.3934	4.9804
	6	0.014445	0.14559	0.1678	0.82832	1.3883	3.0259	17.9374
	7	0.0093642	0.0043877	0.23503	0.14078	5.6004	12.0551	20.7852
	8	0.042432	0.045163	0.13136	1.1432	0.15513	7.733	52.0662
	9	0.031497	0.030422	0.21833	0.46885	0.50191	1.5054	7.073
	10	0.019808	0.019595	0.12494	1.5721	3.5097	4.3163	5.9396

**Table 5:** Target normal error results of image elliptical edge points with noise.

Normal Vector Error (°)		SNR						
		100	90	80	70	60	50	40
**Testing times/** **freq**	1	0.018871	0.1817	0.19367	0.62689	3.3064	7.2458	5.7201
	2	0.045491	0.055941	0.5333	0.93956	1.8872	7.5464	11.0961
	3	0.028009	0.21031	0.4529	1.1693	1.7618	1.2497	10.4465
	4	0.031008	0.04027	0.21542	0.32845	5.1099	20.9333	9.6278
	5	0.041191	0.083118	0.16628	0.085066	2.3522	5.0255	25.7027
	6	0.019423	0.056084	0.23861	1.0037	3.2697	4.6793	6.197
	7	0.027558	0.11894	0.19924	0.89778	3.7449	6.3814	33.0987
	8	0.024983	0.0089671	0.090328	1.8253	3.2571	14.0755	18.0032
	9	0.03971	0.031869	0.26963	0.78701	0.97456	3.0075	30.023
	10	0.037593	0.046394	0.11156	0.66668	4.426	10.3506	12.4495

**Table 6:** Influence of the elliptical edge on space circular target parameters.

	SNR	Center Error/mm	Normal Error (°)
**Traditional** **Algorithm**	100	0.020479419	0.03141138
	90	0.05156101	0.07179895
	80	0.1989628	0.2374018
	70	0.53597785	0.6931265
	60	1.49148405	2.7898965
	50	7.1802945	9.6292235
	40	37.409125	24.113495
**New** **Algorithm**	100	0.020932095	0.031870895
	90	0.056576385	0.076987605
	80	0.2159454	0.2661669
	70	0.52002465	0.7130258
	60	1.5883833	2.783796
	50	6.296056	8.1799965
	40	22.92037	18.808205

**Table 7:** User calibration results.

User	Method Based on Iris Center and Normal Vector	
	Iris Radius/mm	Kappa Angle (°)
1	6.002	5.8392
2	5.276	4.9965
3	6.577	5.0877
4	5.274	5.7297
5	5.736	5.1665

**Table 8:** Comparison of 3D gaze estimation precision.

User	Traditional Method		Proposed Method	
	RMSE ($\varepsilon_{x}$)/°	RMSE ($\varepsilon_{y}$)/°	RMSE ($\varepsilon_{x}$)/°	RMSE ($\varepsilon_{y}$)/°
1	1.675401	1.9473737	1.2055469	1.6732144
2	1.881632	0.565529	1.017873	1.119479
3	2.078221	0.910963	1.639549	1.268943
4	1.85805	0.592964	2.048654	2.204155
5	1.700086	2.283947	1.597281	1.310305
Average	1.838678	1.260155	1.501781	1.515219

## Data Availability

The data presented in this study are available on request from the corresponding author. The data are not publicly available because they have not been ordered and stored in a clear and manageable form.
